# Bile acid bio-nanoencapsulation improved drug targeted-delivery and pharmacological effects via cellular flux: 6-months diabetes preclinical study

**DOI:** 10.1038/s41598-019-53999-1

**Published:** 2020-01-09

**Authors:** Armin Mooranian, Susbin Raj Wagle, Bozica Kovacevic, Ryu Takechi, John Mamo, Virginie Lam, Gerald F. Watts, Momir Mikov, Svetlana Golocorbin-Kon, Goran Stojanovic, Hesham Al-Sallami, Hani Al-Salami

**Affiliations:** 1 Biotechnology and Drug Development Research Laboratory, School of Pharmacy and Biomedical Sciences, Curtin Health Innovation Research Institute, Curtin University, Perth, Western Australia Australia; 2 School of Public Health, Curtin Health Innovation Research Institute, Curtin University, Perth, Western Australia Australia; 3 School of Medicine, Faculty of Health and Medical Sciences, University of Western Australia, Perth, Australia; 4 Lipid Disorders Clinic, Department of Cardiology, Royal Perth Hospital, Perth, Australia; 5 Department of Pharmacology, Toxicology and Clinical Pharmacology, Faculty of Medicine, University of Novi Sad, Novi Sad, Serbia; 6 Department of Pharmacy, University of Novi Sad, Novi Sad, Serbia; 7 Faculty of Technical Sciences, University of Novi Sad, Novi Sad, Trg Dositeja Obradovica 6, 21000 Novi Sad Serbia; 8 School of Pharmacy, University of Otago, Dunedin, New Zealand

**Keywords:** Clinical pharmacology, Drug delivery

## Abstract

The antilipidemic drug, probucol (PB), has demonstrated potential applications in Type 2 diabetes (T2D) through its protective effects on pancreatic β-cells. PB has poor solubility and bioavailability, and despite attempts to improve its oral delivery, none has shown dramatic improvements in absorption or antidiabetic effects. Preliminary data has shown potential benefits from bile acid co-encapsulation with PB. One bile acid has shown best potential improvement of PB oral delivery (ursodeoxycholic acid, UDCA). This study aimed to examine PB and UDCA microcapsules (with UDCA microcapsules serving as control) in terms of the microcapsules’ morphology, biological effects *ex vivo*, and their hypoglycemic and antilipidemic and anti-inflammatory effects *in vivo*. PBUDCA and UDCA microcapsules were examined *in vitro* (formulation studies), *ex vivo* and *in vivo*. PBUDCA microcapsules exerted positive effects on β-cells viability at hyperglycemic state, and brought about hypoglycemic and anti-inflammatory effects on the prediabetic mice. In conclusion, PBUDCA co-encapsulation have showed beneficial therapeutic impact of dual antioxidant-bile acid effects in diabetes treatment.

## Introduction

Understanding the link between insulin-resistance, prediabetes and Type 2 diabetes (T2) is anticipated to facilitate better ability to design new interventions in order to control the fast growing epidemic of diabetes. The link encompasses multiple physiological disturbances including obesity. In a review by Qatanani, M. and Lazar, M.A, the authors have examined specific links between insulin resistance and visceral adiposity and excess fat accumulation in blood and tissues^1^. They found that there is a direct correlation between the amounts of lipid represented by biomarkers such as total cholesterol, triglycerides and noneesterified fatty acids (NEFA), and the extent of insulin-resistance and rate of prediabetes development. One of the possible underlying mechanisms to insulin-resistance and prediabetes, has been hypothesized to be oxidative stress and inflammation^2–6^. Oxidative stress and local and systemic inflammation have been shown to be contributing factors in development of insulin-resistance, prediabetes and eventually T2D. Oxidative stress and inflammation have also been linked to worsening of diabetic symptoms and long-term prognosis^7,8^. In addition, diabetes-inflammation has been associated with lipid dysregulation, visceral adipose tissue accumulation and insulin-resistance. Karpe, F. *et al*.; have shown direct association between levels of inflammatory cytokines, with development of visceral fat accumulation, insulin-resistance, and prediabetes^9^. Studies published by our laboratory have also demonstrated direct links between concentrations of proinflammatory cytokines: TNF-α, IFN-γ, IL-1β and IL-6 levels with pancreatic β-cell functions and insulin secretions at normoglycemia and hyperglycaemic states^4,10–14^. Overall, better control over prediabetes and diabetes development may be achieved by new and optimised therapeutics which not only target glycemia but also ameliorate or even prevent the underlying inflammation. New and optimised therapeutics can incorporate and encapsulate multiple drugs (e.g. including potent antioxidants such as probucol “PB”), which synergistically target oxidative stress and inflammation in the diabetic hyperglycaemic state.

Of the endogenous bile acids, the tertiary bile acid ursodeoxycholic acid (UDCA) has been shown to be the most potent anti-inflammatory and anti-apoptotic bile acid with significant cell protective properties with its mechanism of action being correlated with its cellular uptake by muscle and β-cells^15–20^. Tsuchida, T., *et al*.; have shown that chronic oral administrations of UDCA in insulin resistant animals have been associated with cardio-metabolic improvement as a result of its cellular uptake^21^. Other studies involving human clinical trials have shown that UDCA is well tolerated and exerts positive effects on glucose and energy homeostasis^22–24^. Many bile acids such as chenodeoxycholic acid (CDCA) and lithocholic acid (LCA) have also shown to be affected by the development and progression of insulin-resistance and diabetes, as well as associated-inflammation^4,5,25,26^. To date and to the best of our knowledge, no studies have examined PBUDCA pharmaceutical properties, their effects on both pancreatic β-cells and muscle cells, their cellular uptake, permeation and interaction with the ABC protein transporters Multi-Resistance Associated Proteins (MRP) 1, 2 and 3, and the preclinical long-term efficacy of these microcapsules on glucose regulation, lipid and inflammatory profiles, as well as their effects on the bile acid profile, in a mouse model of insulin-resistance.

Thus, the aim of this study was to develop microcapsules incorporating PB and UDCA and examine the morphological, physical and chemical compatibility of the microcapsules (*in vitro*), their effects on pancreatic and muscle cells (*ex vivo*), cellular uptake and flux of PB and UDCA, and their potential hypoglycaemic, antilipidemic and antiinflammatory effects in a mouse model of prediabetes, given oral microcapsules for 6 months (*in vivo*). PB oral uptake and concentrations in plasma, tissues (ileum, liver, brain, heart, pancreas, and kidney) and faeces will be measured. The bile acids CDCA, LCA and UDCA concentrations in plasma, tissues and faeces will also be measured. The study is a continuation of ongoing work in our laboratory examining the preclinical efficacy of dual ingredient antioxidant-bile acid microcapsules in diabetes mellitus with published hypoglycaemic effects of controlled groups^3,11,14,27–35^.

## Results

### Microencapsulation fabrication, and stability/shelf life *in vitro* studies

**Figure 1:**
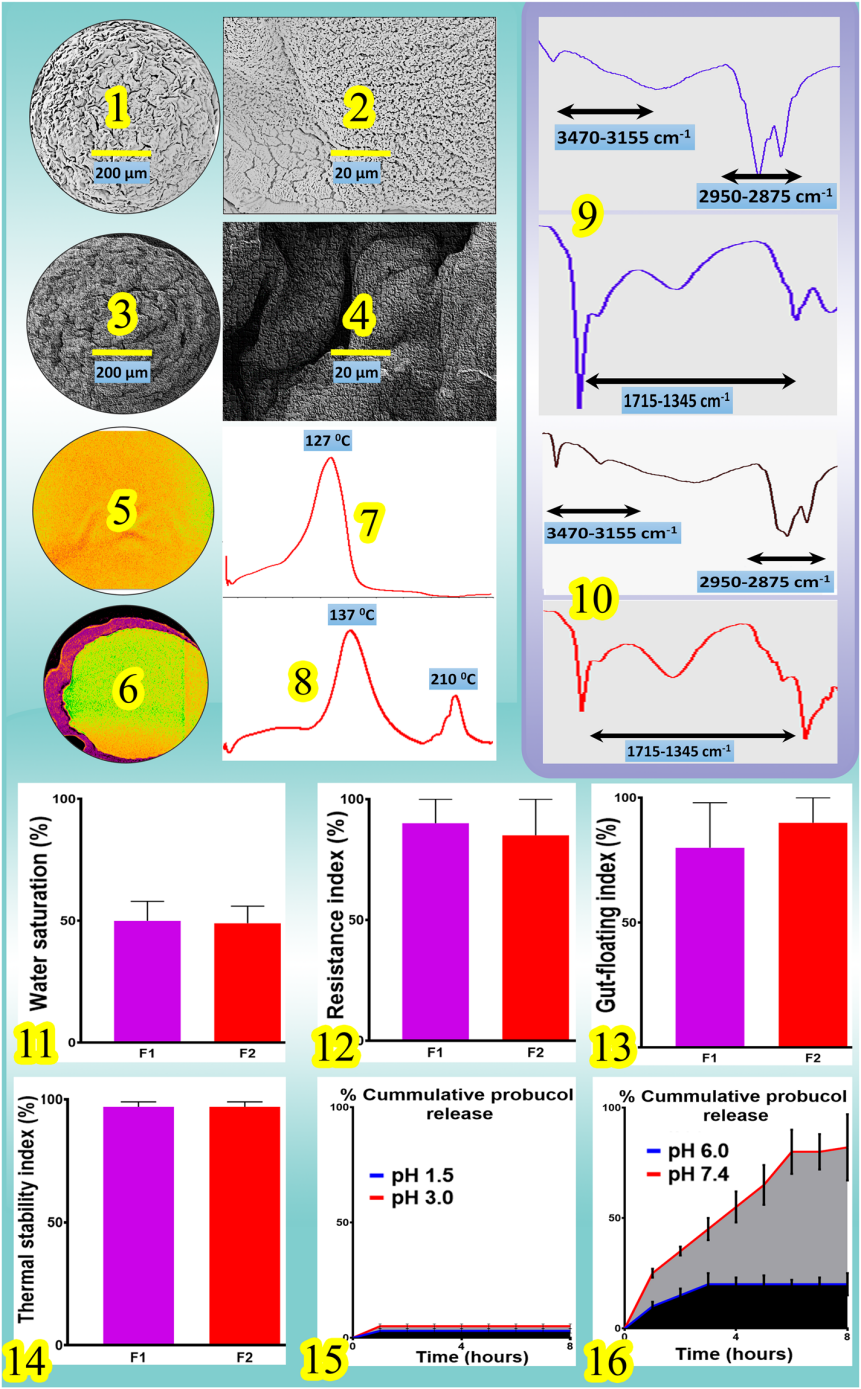
SEM (1–4), Micro-CT (5–6), DSC (7–8), FTIR-IR (9–10), water saturation index (11), microcapsules resistance index (12), PB release at pH 1.5, 3, 6 and 7.4 (13–14), gut-floating index (15) and thermal stability index (16) of UDCA and PBUDCA microcapsules respectively. Data are mean + /− SEM (n = 3).

Stability and shelf life *in vitro* studies showed stable microcapsules over a period of 2 weeks, at temperatures <40 °C and a relative humidity of <35%. In addition, Fig. 1 shows the SEM micrographs (UDCA: 1–2, PBUDCA: 3–4), Micro-CT (UDCA: 5, PBUDCA: 6), DSC spectra (UDCA: 7, PBUDCA: 8), FTIR (UDCA: 9, PBUDCA: 10), water saturation index (UDCA: 11, PBUDCA: 12), gut-floating index (13), thermal stability index (14), and PB cumulative drug release at pH 1.5 and 3 (15) and PB cumulative drug release at pH 6.0 and 7.4 (16) of F1 (UDCA microcapsules) and F2 (PBUDCA microcapsules). SEM micrographs showed similar shape and size with some variation between F1 and F2 in terms of F1 having more solid surface with less cracked and pores, which suggests that F2 has porous outer surface, compared with F1. Micro-CT images showed distinct outer surface of the bile acid containing microcapsules, suggesting bile acid accumulation on the surface or outer layers of the microcapsules. DSC and FTIR spectra showed consistent thermal and chemical capacities for both types of microcapsules, suggesting thermo-chemical stability of microcapsules constituents, while water saturation, resistance, gut-floating and thermal indices showed consistency in both F1 and F2 microcapsules with PB release demonstrating pH targeted delivery (Fig. 1).

### *Ex vivo* studies

**Figure 2:**
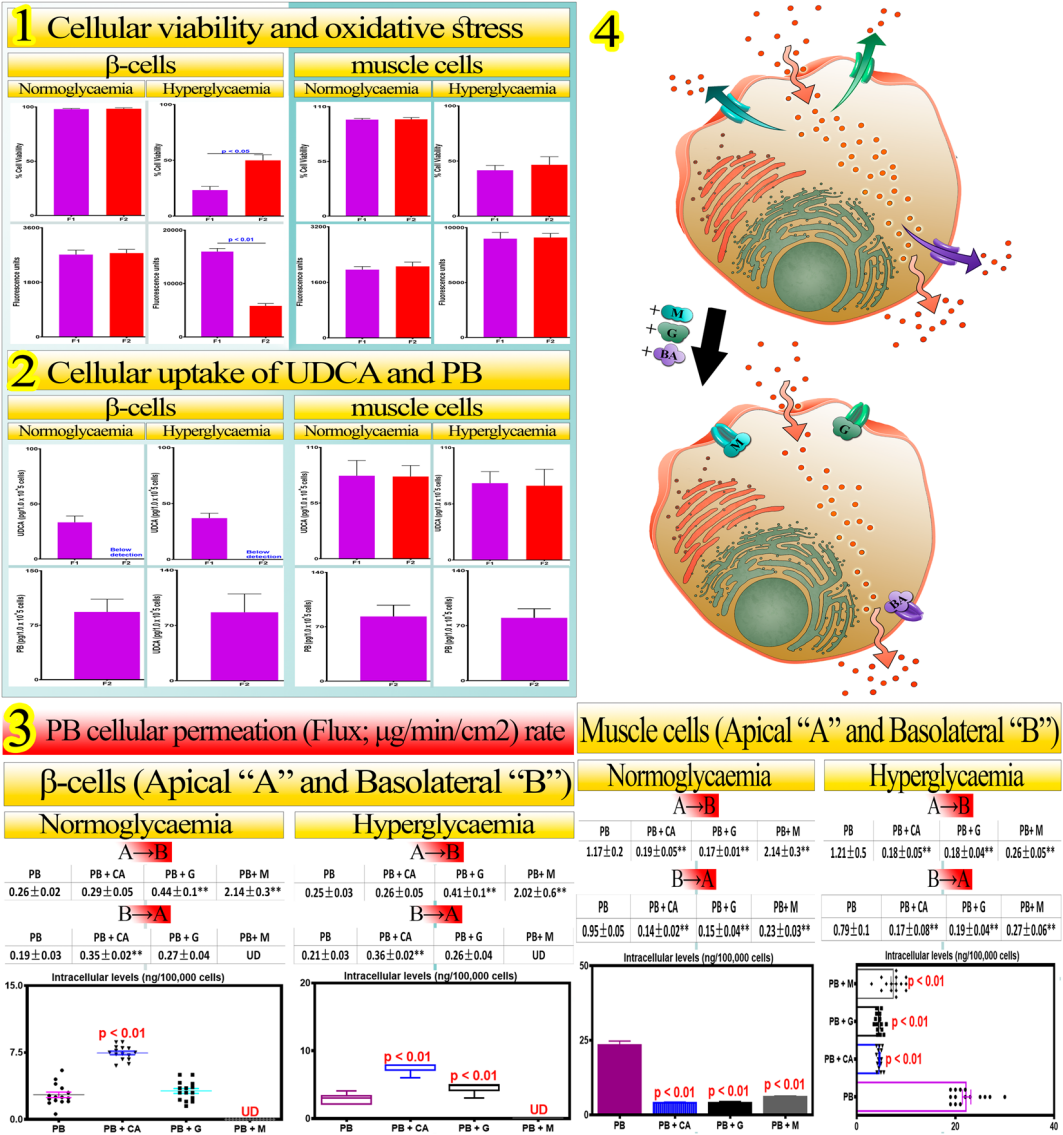
Cellular viability and oxidative stress (1), cellular uptake of UDCA and PB (2), PB unidirectional cellular permeation (flux) and cellular amount retained (3), and cellular efflux protein-transporters competitive inhibition (4), in β-cell and muscle cell, at the normoglycaemic and the hyperglycaemic states. F1: UDCA microcapsules and F2: PBUDCA microcapsules. G-gliclazide; M-metformin; CA-cholic acid. Data are mean + /− SEM (n = 3).

Figure 2 shows effects of PB and PBUDCA microcapsules on cell viability and oxidative stress (1), their cellular uptake (2), and cellular permeation (3) and efflux protein-transporters effects (4), at normoglycaemic (healthy) and hyperglycaemic (diabetic) states, using two cell types, β-cells and muscle cells.

**Figure 3:**
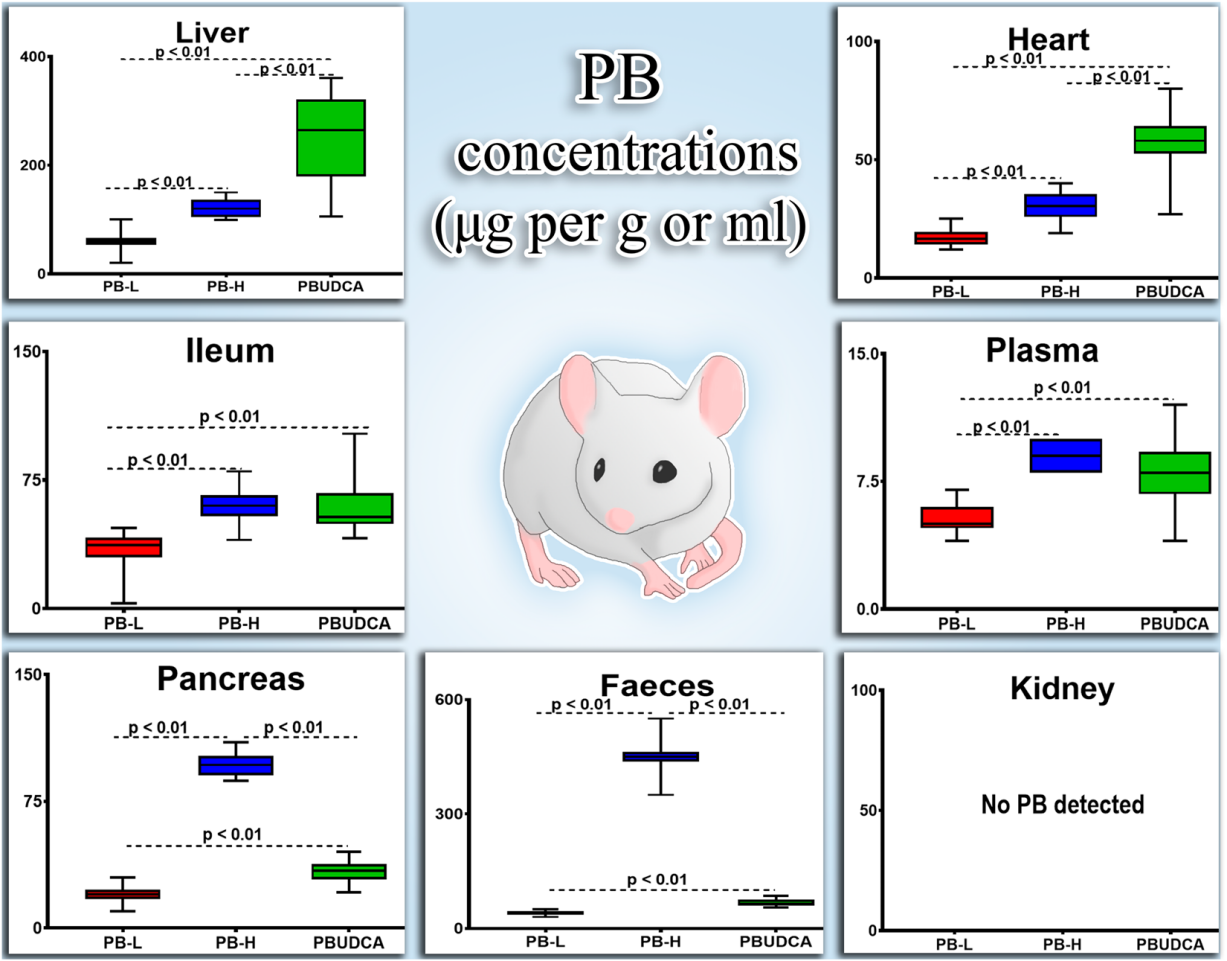
PB concentrations in serum, tissues and faeces. PB-L: low dose probucol, PB-H: high dose probucol and PBUDCA: probucol-ursodeoxycholic acid microcapsules. Data are mean ± SEM. **p < 0.01, compared with HFD.

**Figure 4:**
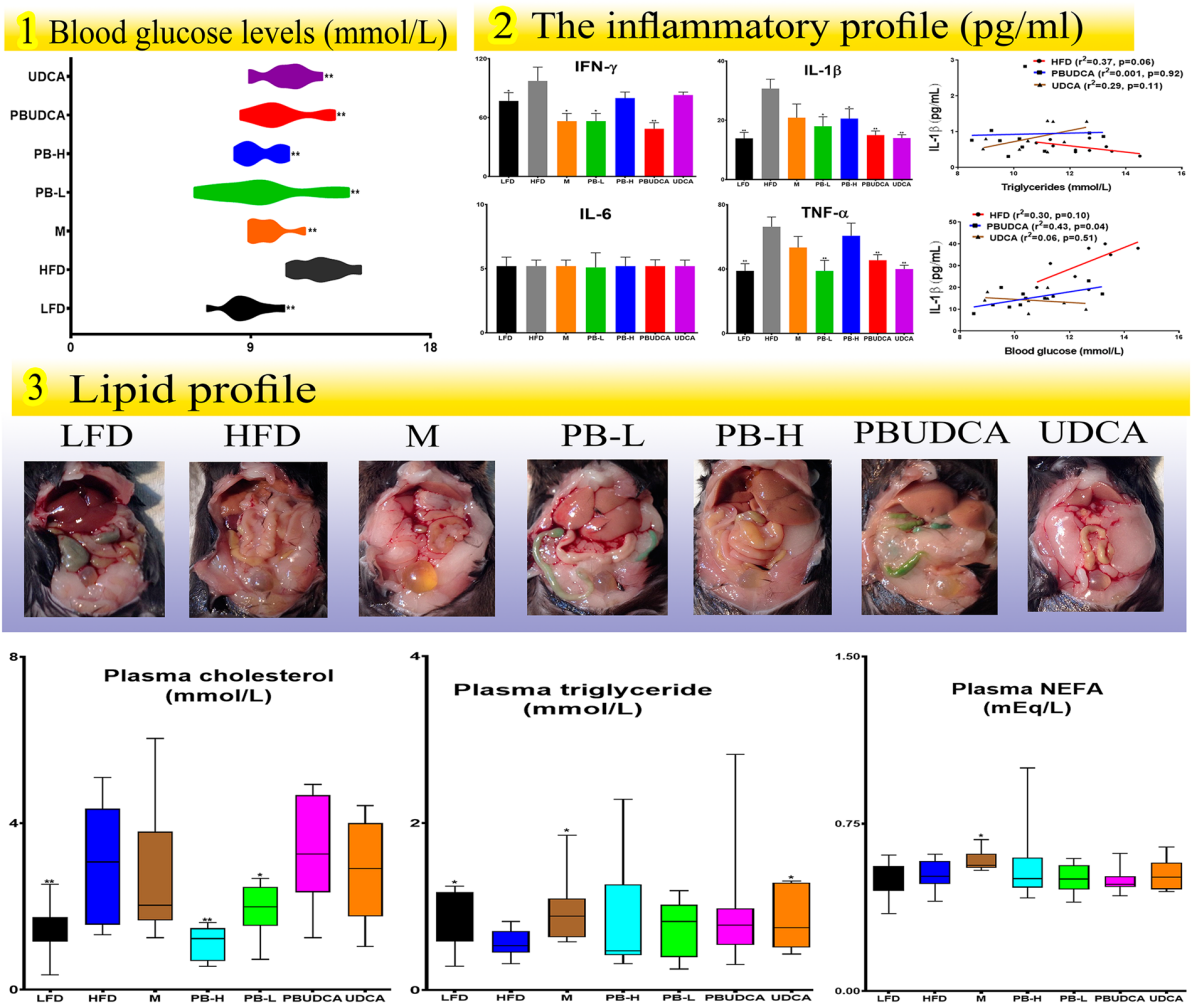
Blood glucose levels (1), plasma inflammatory profile and Linear Regression analyses (2), and plasma lipid profile (3). LFD: low-fat diet, HFD: high-fat diet, M: metformin, PB-L: low dose probucol, PB-H: high dose probucol, PBUDCA: probucol-ursodeoxycholic acid microcapsules, and UDCA: ursodeoxycholic acid microcapsules. Data are mean ± SEM. *p < 0.05 and **p < 0.01 com*p*ared with HFD.

Cellular viability of pancreatic β-cells and muscle cells were unchanged in normoglycaemic conditions when exposed to F1 and F2, while in hyperglycaemic conditions, β-cell viability was improved by F2 exposure which showed lower fluorescence (Figs. 1–2). Cellular uptake of UDCA was higher when the β-cells were exposed to F1 compared with F2 (normoglycaemic and hyperglycaemic states) while UDCA and PB cellular uptake remained unchanged when cells were exposed to F1 or F2 in normoglycemic and hyperglycaemic states (Fig. 2–2). In normoglycemia and hyperglycaemia, PB (A → B) unidirectional cellular permeation (flux) was higher when combined with G or M, and higher with CA (B → A) with M, and combination and intracellular concentrations of PB showing highest levels when with CA (Figs. 2–3) with hypothesized mechanisms of PB cellular uptake illustrated (Figs. 2–4).

### *In vivo* studies

Figure 3 shows the PB levels, from PB-L, PB-H and PBUDCA groups, in liver, ileum, pancreas, faeces, plasma, heart and kidney.

PB was detected in all tissues and faeces except kidneys. Compared with PB-L, both, PB-H and PBUDCA resulted in increased PB levels in plasma, ileum, liver, brain, heart, pancreas and faeces with higher PB levels in PB-H plasma, tissues and faeces, compared with PB-L. PB-L and PBUDCA (with same dose of PB) resulted in higher PB levels in plasma and tissues with PB-L, implicating MRP3 inhibition. Levels of PB in pancreas were low in PB-H and liver levels showed higher PB concentrations.

Figure 4 shows blood glucose levels (1), the inflammatory biomarkers IFN-γ, IL-1β, IL-6 and TNF-α (2), and the lipid biomarkers total cholesterol, triglyceride and NEFA levels (3) at the end of the experiment and association between IL-1β with blood triglycerides and blood glucose levels among HDF, PBUDCA and UDCA groups.

Compared with control (HFD), all treatments reduced blood glucose (Figs. 1–4), while reduction of inflammatory biomarkers was not consistent among all measured cytokines and by all treatments with PB-L, PBUDCA and UDCA treatments exerting most antiinflammatory effects and PBUDCA showing significant correlation with IL-1β and triglycerides control (Figs. 2–4). Effects of treatment on lipid profile and plasma cholesterol was potent and evident with PB-H treatment, while other treatments had little/no effects on the lipid profile (Figs. 3–4).

### The bile acid profile

**Figure 5:**
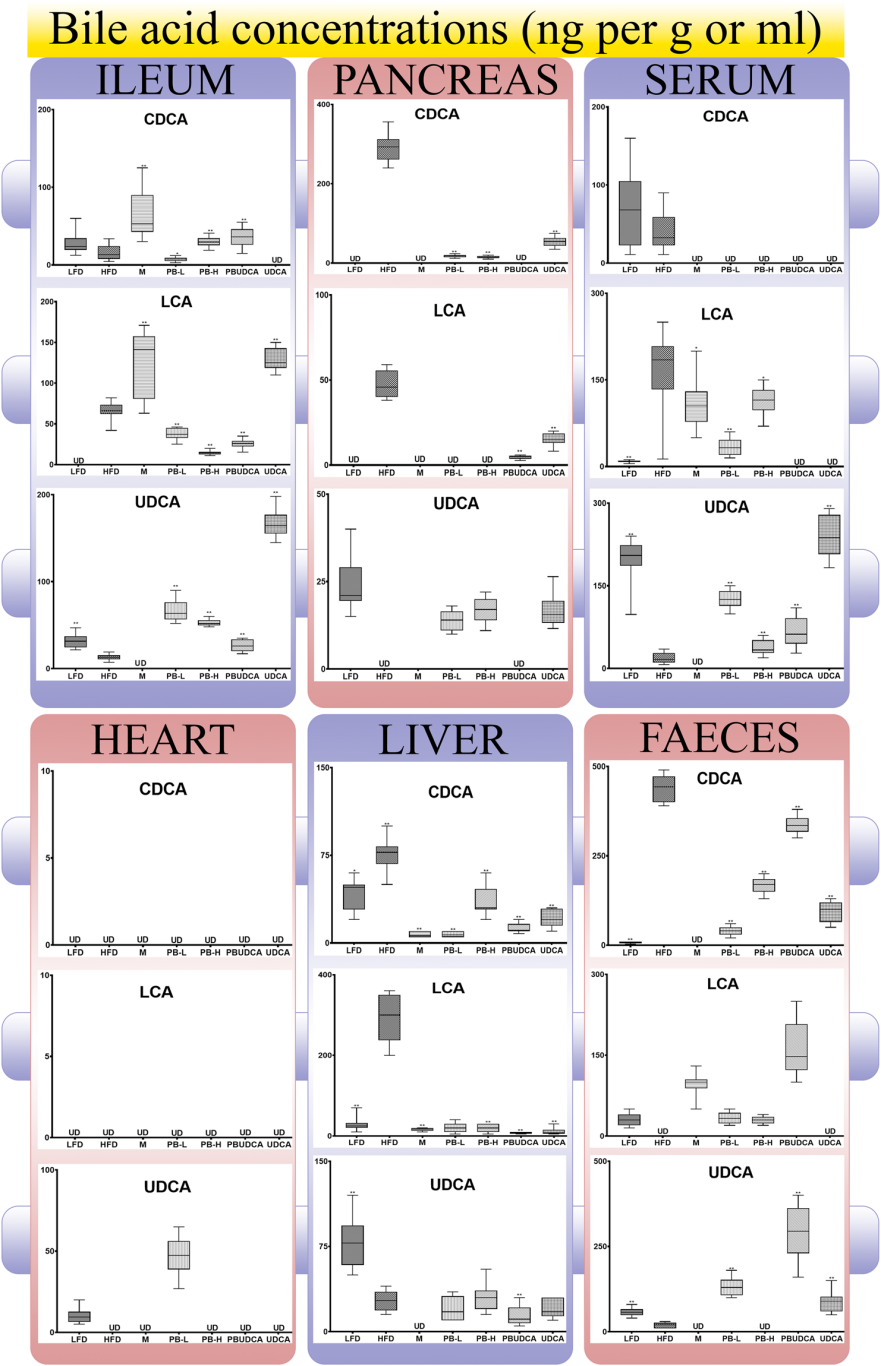
Bile acid concentrations in serum, tissues and faeces. LFD: low-fat diet, HFD: high-fat diet, M: metformin, PB-L: low dose probucol, PB-H: high dose probucol, PBUDCA: probucol-ursodeoxycholic acid microcapsules, and UDCA: ursodeoxycholic acid microcapsules. Data are mean ± SEM. *p < 0.05 and **p < 0.01 com*p*ared with HFD.

Figure 5 shows levels of the primary bile acid CDCA, the secondary bile acid LCA and the tertiary bile acid UDCA in tissues, serum, and faeces.

The bile acid profile is significantly influenced by various treatments with all groups demonstrating significant alteration of bile acid concentrations at the end of the experiment, in ileum, pancreas, serum, heart, liver and faeces. There was significant undetected levels of bile acids in heart suggesting lowered uptake as a result of diabetes development. There was reduction in levels of the primary bile acid, CDCA in serum and due to M, PB-L, PB-H, PBUDCA and UDCA treatments but levels of secondary and tertiary bile acids (LCA and UDCA) were detected in all treated groups suggesting feedback mechanisms compensating for primary bile acid reduction, which was also noticed in heart tissues (Fig. 5).

## Discussion

Figure 1 suggests that there was no significant change in morphology, size, multi-surface features in terms of UDCA and PB or UDCA particle distribution within the layers, or surface topographical features between UDCA and PBUDCA microcapsules, despite reduced porosity in F2, which suggests that PB or UDCA presence in microcapsules did not alter topographic properties, multi-layered surface composition, or compromise microcapsules’ shape or size. This also suggests that the microencapsulation method was robust and resulted in uniform microcapsules regardless of PB or UDCA presence. DCS and FTIR analyses of UDCA and PBUDCA microcapsules showed small but distinct variation in wavelengths’ intensity and range as well as similar melting points suggesting stability of ingredients during the encapsulation process. Specifically, in PBUDCA microcapsules compared with UDCA microcapsules, there was a new peak in the 3470–3155 cm^−1^ region as well as alternations in peak-bond activity in the 1715–1345 cm^−1^ region. The new peak and alterations are likely attributed to C-H stretch in the alkane bonds and O-H stretch in the phenol groups within PB’s molecular structure as well as C-C stretch within the aromatic rings of PB (occurring around 1420 cm^−1^) and C-O stretch from the phenol groups of PB (occurring around 1300 cm^−1^)^14,27,29,31^. Such changes in the FTIR and DSC measurements suggests new bond-formation as a result of PB presence, but chemical compatibility was maintained as no bond-activity has completely disappeared nor the new spectra resulting from PB presence was completely different hence a less likely hood of chemical degradation or drug alteration within the microcapsules. This is consistent with our previous studies that showed compatibility between different bile acids and PB presence within the microcapsules^11,14,28,33^. Morphological and compatibility results suggest that PB presence in UDCA microcapsules did not affect the physical characteristics of the microcapsules. Changes in moisture contents (water saturation), physical resistance to stress, floating and thermal stability showed no significant difference between the two microcapsules suggesting that the osmotic stability, mechanical resistance, buoyancy and heat resistance properties remained similar. Results suggest that the presence of PB did not change the physico-chemical properties of the microcapsules and intactness of microcapsules remained consistent, postmicroencapsulation, regardless of PB presence. This supports the notion that our microencapsulation methods was robust and maintained uniformity and preserved the physical properties and structural integrity of the microcapsules. This is also in line with other research groups that showed positive effects of bile components on intestinal drug delivery. Hunt, G.R. and I.C. Jones showed improvement in intestinal drug delivery, to liposomal delivery, by using the bile salts, glycocholate and glycodeoxycholate^36^. However, despite the fact that both microcapsules, PBUDCA and UDCA showed good morphological and physical properties, their impact on cell viability and oxidative stress need to be investigated in order to elucidate beneficial effects at the cellular level. In addition, UDCA potential synergistic effects when combined with PB are likely to be associated with its cellular uptake as UDCA is endogenously produced and metabolised intracellularly.

Cellular protective effects indicative by higher cell viability of PBUDCA microcapsules might be the result of its direct inhibitory effects on oxidative stress of β-cells at hyperglycaemic state, by PB and UDCA, which resulted in normalisation of free radicals and subsequent protection of β-cells from radical damage. Hence, efflux in the presence of PBUDCA resulted in modulation of PB uptake likely by UDCA co-encapsulation affecting specific protein transporters. This is consistent with our studies showing protective antioxidant effects of PB-bile acid microcapsules^3^. This is also consistent with other research groups that have shown strong β-cell protective effects of UDCA or its metabolites. Engin, F. *et al*.; have shown that a conjugated UDCA exerted antiinflammatory effects and reduced loss of β-cell viability and reduced cell apoptosis through its positive effects on unfolded protein response and its mediators^37^, while Kim, JY *et al*.; have shown that bile acids are taken up intracellularly into pancreatic cells and influence cell apoptosis by affecting cellular Ca^2+^ signalling pathways^38^.

The similar amount of UDCA absorbed by β-cells at normal and hyperglycaemic states suggests that UDCA cellular uptake was independent of glucose concentrations or the glycaemic state. UDCA cellular uptake by muscle cells was significant and independent of glucose levels or the presence of PB in the microcapsules, while UDCA cellular uptake by β-cells was only significant from UDCA microcapsules, and not PBUDCA microcapsules, which suggests that PB selectively inhibited UDCA uptake by β-cells. One possible way by which PB inhibited UDCA uptake by β-cells, was by competitively inhibiting protein-transporters responsible for UDCA uptake into the cells. In one study, Geier, A. *et al*.; have demonstrated that the bile acid UDCA is a substrate of multiple protein transporters in liver such as ABC-transporters and multidrug resistance associated protein 3 (MRP 3) and inhibiting these proteins may affect UDCA cellular uptake^39^. In many other studies, PB has been hypothesized to be capable of competitive inhibition of many protein-transporters responsible for bile acid uptake in liver and pancreas. Rinninger, F *et al*.; have showed strong association between PB cellular transport and the scavenger receptor BI^40^. In another study, Ma, Q, *et al*.; have revealed that the protein transporter which belongs to the ABC transporters, ABCB1, has strong affinity for PB molecules, and ABC transporters are known to also target bile acids^41^. Accordingly, at hyperglycaemic state, PBUDCA microcapsules improved β-cell viability, but impaired UDCA cellular uptake, while both microcapsules exerted similar effects on muscle cells, in terms of viability, oxidative stress and UDCA cellular uptake. The presence of PB exerted favourable cell protective and antioxidant effects on β-cells. Thus, PB concentrations in plasma and tissues should provide an insight on its cellular uptake, as well as its impact on bile acid profile, glucose levels, lipid profile and the inflammatory response.

Compared with UDCA microcapsules, PBUDCA microcapsules resulted in higher β-cell viability, and lower oxidative stress at hyperglycaemic state, while neither PBUDCA nor UDCA microcapsules had effects on C2C12 cells viability or oxidative stress levels (Figs. 1–2). UDCA uptake by β-cells from UDCA microcapsules was significantly high at both glucose levels, while no UDCA uptake was detected from PBUDCA microcapsules. UDCA uptake by muscle cells was significant and consistent at both glucose levels and irrespective of PB incorporation into microcapsules. PB uptake was significant from PBUDCA microcapsules, at both glucose levels (Fig. 2–2). PB’s cellular permeation (flux) and cellular retention were modified (Figs. 2–3) by selective substrates of certain ABC-efflux protein transporters (multi-resistance proteins ‘MRP’ 1, 2 and 3) which may provide information on mechanisms of PB cellular uptake (Figs. 2–4) relevant to its physiological effects on viability and oxidative stress (Fig. 2: 1 & 2). The addition of gliclazide (G; a selective substrate for MRP2^42^), metformin (M; a selective substrate for “MRP1”^43^) and the bile acid cholic acid (CA; a selective substrate for MRP3^44^) resulted in changes in PB unidirectional apical to basolateral and basolateral to apical flux and subsequent cellular retention in β and muscle cells. The changes of PB flux and cellular retention were possibly via substrate-competitive inhibition by BA, G and M, and these effects were similar at the normoglycaemic versus the hyperglycaemic states, and different when comparing these effects on β-cells versus muscle cells (Table 1). Changes caused by BA, G and M on PB cellular uptake and retention suggest involvement of MRP1, MRP2 and MRP3 in PB cell delivery. The absence of direct and significant effects of hyperglycaemia on PB cell delivery suggests that development of hyperglycaemia and insulin-resistance do not directly affect PB oral uptake or the amount of PB permeating or being retained by cells. In β-cells, MRP3 inhibition resulted in no change of PB unidirectional apical-to-basolateral flux while increasing unidirectional basolateral-to-apical flux and cell retention suggesting that PB has substrate affinity for MRP3 and hence inhibiting MRP3 efflux of PB resulted in constant A→B permeation with significant increase in B→A and cell retention due to increased uptake from the basolateral side of the cells. MRP2 inhibition resulted in increased PB unidirectional apical-to-basolateral flux while having no effects on basolateral-to-apical flux and cellular retention suggesting that PB has substrate affinity for MRP2 on the apical side while its MRP3 efflux is maintained. MRP1 inhibition resulted in increased apical-to-basolateral influx, with reduction in basolateral-to-apical flux and cellular retention suggesting that either inhibiting MRP1 only increased apical-to-basolateral flux with no up-regulation of other efflux transporters transporting cellular PB, or there are other PB efflux transporters, on the basolateral side, that were inhibited by M addition^45,46^.

**Table 1:** Summary of the influence of incorporation of a bile acid (BA), gliclazide (G) and metformin (M) on probucol (PB) cellular permeation (flux) and cellular retention using two cell types (β and muscle cells) at the normoglycaemic and hyperglycaemic states.

	Normoglycaemic state			Hyperglycaemic state		
**β-cells**						
PB	+BA (−MRP3)	+G (−MRP2)	+M (−MRP1)	+BA (−MRP3)	+G (−MRP2)	+M (−MRP1)
A→B	±	↑	↑	±	↑	↑
B→A	↑	±	↓	↑	±	↓
Cellular retention	↑	±	↓	↑	±	↓
**Muscle-cells**						
PB	+BA (MRP3)	+G (−MRP2)	+M (−MRP1)	+BA (MRP3)	+G (−MRP2)	+M (−MRP1)
A→B	↓	↓	↑	↓	↓	↓
B→A	↓	↓	↓	↓	↓	↓
Cellular retention	↓	↓	↓	↓	↓	↓

±indicates no significant net effect, ↑ indicates increase and ↓ indicates decrease levels, based on Fig. 2–2 measurements.

In muscle-cells, MRP3 inhibition resulted in reduction in both, apical-to-basolateral and basolateral-to-apical unidirectional fluxes as well as cellular retention suggesting that MRP3 inhibition brought about reduction in the amount of PB passing through the cells and the overall cellular uptake of PB, likely via either suppression of PB permeation or optimisation of PB overall cellular efflux. Similar to MRP1 inhibition, MRP2 inhibition resulted in reduction in apical-to-basolateral and basolateral-to-apical unidirectional fluxes and less PB cellular retention, and hence a significantly different response in muscle cells compared with pancreatic β-cells suggesting that ABC-efflux protein expression is significantly different between both types of cells, and this is consistent with the literature^47,48^. Similar to MRP1 inhibition in β-cells, muscle-cells MRP1 inhibition resulted in higher apical-to-basolateral flux with reduced basolateral-to-apical flux as well as cellular retention, which suggests PB permeation and retention remain constant between both types of cells and hyperglycaemia did not affect that, although PB apical-to-basolateral flux was reduced indicating direct effects of diabetes on expression and functionality of apical MRP1 in muscle cells. Diabetes-associated alteration of ABC efflux transporters’ expression and functionalities are consistent with the literature^49,50^ suggesting that diabetes development and progression may have detrimental effects on the functions of selective ABC-efflux transporters and result in variation of drug uptake and absorption in diabetic patients (Table 1).

It is worth stating that there is some ambiguity in the literature regarding selectivity of drugs to specific ABC-efflux transporters and the possibility with multiple simultaneous selectivity and various effects of diseases on expression and functionalities of these transporters. Hence, although we maintain that our MRP1, MRP2, and MRP3 substrates/inhibitors are selective based on our work and others, tissue specific competitive-inhibition remains debatable and a study limitation, particularly when other transporters are also prominent e.g. organic anion transporting proteins and organic cation transporter polypeptide for bile acids^42–44,51,52^.

PB was detected in all analysed samples except kidneys, which likely due to its high lipophilic properties and, thus, excretion is mostly by liver rather than kidney, and this is consistent with the literature^53,54^. Compared with PB-L, both, PB-H and PBUDCA resulted in increased PB levels in plasma, ileum, liver, brain, heart, pancreas and faeces. The higher concentrations of PB in PB-H plasma, tissues and faeces, compared with PB-L, was expected and suggests that PB absorption does not reach saturation levels after PB-L, potentially via MRP1 or MRP2 and MRP3 involvement. Despite PB-L and PBUDCA containing the same amount of PB, PBUDCA resulted in higher PB concentrations in plasma and all tissues analysed compared with PB-L, which suggests that incorporation of PB into UDCA-microcapsules enhanced its absorption and tissue accumulation as well as its concentrations in systemic circulation potentially via UDCA inhibition of MRP3 resulting in net enhancement of PB cellular permeation. Pancreas levels of PB remained low with regard to PB-H while liver achieved higher PB levels, possibly due to cellular uptake being limited in the pancreas, but not hepatocytes, hence higher liver accumulation of PB when given encapsulated with UDCA. The improved absorption of PB with microencapsulation is also consistent with findings from other research groups. Fukami, T. *et al*.; have shown that special delivery methods such as nanotechnology can significantly enhance PB’s absorption and cellular permeation^55^, while Zhang, Z. *et al*.; have demonstrated that using hybrid of surfactants which form negatively charged particles can further enhance the oral delivery of PB^56^. With the significant increase in PB concentrations in plasma and tissues, as a result of PB-H or PBUDCA microcapsule treatments, effects on blood glucose, inflammation and lipid profiles by different treatments, should provide insight on association between PB levels, glycaemic control and pharmacological effects on inflammation and lipid levels, in our insulin-resistance animal model.

With regard to treatments’ effects on blood glucose, inflammation and lipid profile (Fig. 4), All treated groups showed significant reduction in blood glucose levels compared with control (with similar magnitudes) which suggests that PBUDCA microcapsules did not produce any additional hypoglycaemic benefits compared with other groups. The similar hypoglycaemic effects between PB-L, PB-H and PBUDCA suggest that the hypoglycaemic effect is independent of PB dosing or its oral delivery formulation, and may relate to treatments’ effects on inflammatory or lipid profiles (Fig. 4: 2 & 3). Results from plasma levels of IFN-γ, IL-1β, TNF-α and IL-6 showed that all treatment groups exerted anti-inflammatory effects through reducing at least one of the four pro-inflammatory biomarkers (Table 2). Although none of the treatment significantly changed IL-6 levels, PB-L and PB-UDCA treatments exerted the most antiinflammatory effects via reducing three pro-inflammatory biomarkers IFN-γ, IL-1β, and TNF-α. In addition, PB-L, and PBUDCA also brought about the greatest TNF-α lowering effect similar to that of M. There was weak association between PBUDCA effects on IL-1β and lowering of blood glucose levels which suggests that reducing inflammation assisted with the hypoglycaemic effects, although exact cellular mechanisms remained to be investigated. In addition, the results showed PBUDCA having the greatest anti-inflammatory effects and this may be due to the combined syncretistic biological impact of the potent anti-oxidant PB coupled with the anti-inflammatory bile acid UDCA^26,57,58^. Plasma lipid profiles and visceral fat depositions could impact on the glycaemic and the inflammatory profiles.

**Table 2:** Summary of the levels of various glycaemic, inflammatory and lipid profiles among treated groups compared with control (HFD).

Treatments	Glycaemic profile	Inflammatory profile				Lipid profile		
	Blood glucose (mM)	IFN-γ (pg/ml)	IL-1β (pg/ml)	IL-6 (pg/ml)	TNF-α (pg/ml)	Cholesterol (mM)	Triglycerides (mM)	NEFA (mEq/L)
LFD	↓	↓	↓	±	↓	↓	↑	±
M	↓	↓	±	±	±	±	↑	↑
PB-L	↓	↓	↓	±	↓	↓	±	±
PB-H	↓	±	↓	±	±	↓	±	±
PB-UDCA	↓	↓	↓	±	↓	±	±	±
UDCA	↓	±	↓	±	↓	±	↑	±

±indicates no significant net effect, ↑ indicates increase and ↓ indicates decrease levels, based on Fig. 4 measurements.

Visual examination of the visceral fats showed no visible difference in colure, shape or texture of visceral tissues/fat amongst the groups although such examination was not histopathological or immunophysiological, and hence was considered complementary to the lipid profile analyses. The lipid profile analyses showed that only PB-L and PB-H significantly lowered cholesterol and there was an increase in the triglyceride and NEFA levels with M and triglyceride with UDCA treatments. In addition, none of the treatment groups except M, changed NEFA levels (Table 2). The results of the lipid profile suggest that M has increased triglycerides and NEFA unfavourably affecting the lipid profile, which is consistent with published studies^59^. The lipid-regulatory effects of PB-L and PB-H are expected since PB is a lipid-lowering drug, while UDCA bringing about an increase in triglycerides levels suggests that UDCA administration resulted in negative feedback mechanisms on bile acid synthesis and cholesterol catabolism resulting in higher levels of triglyceride, and modulation of the bile acid profile (Fig. 5).

With regard to the concentrations of CDCA, LCA and UDCA in tissues, serum and faeces, Compared with HFD, all treatments resulted in reduced CDCA plasma concentrations, which suggests direct interference of treatments with either cholesterol catabolism to primary bile acids or accelerated primary bile acid metabolism to secondary bile acids. Ileal CDCA concentrations in LFD were similar to HFD which is consistent with serum levels, and they were higher in M, PB-H and PBUDCA groups while there were not detected in UDCA group suggesting treatments effects on bile acid gut metabolism and ileal cellular uptake. In the pancreas, CDCA levels were the highest in the HDF group and were reduced in all other groups suggesting significant alteration of pancreatic tissue accumulation of the primary bile acid brought about by various treatments that possibly altered cholesterol catabolism and total cholesterol available (Fig. 4) or uptake of metabolised bile acid via gut enterocytes’ efflux transport (Fig. 2). Heart tissue showed no presence of CDCA levels, while in the liver, HFD treatment increased CDCA levels and all other various treatments reduced CDCA levels suggesting reduction in CDCA production and cholesterol catabolism in hepatocytes and possibly inhibiting the enterohepatic recirculation processes. The reduction of CDCA in the liver by various treatments may be associated with increased hepatocytic concentration (Fig. 3) due to possible cellular efflux inhibition of MRP1, MRP2 or MRP3 by treatments and CDCA accumulation being their substrate (Fig. 2). High CDCA levels in faeces due to HFD is consistent to its increase levels in ileum and their reduced levels by treatments. LCA is considered a potent bile acid and has been associated in the literature with cytotoxicity and inflammation^60–62^. Serum LCA levels were elevated in the HFD group and reduced by treatments. Ileal levels were undetected in the LFD group, significantly high in the HFD group and compared with the HFD group, M and UDCA treatments increased that further while PB treatments reduced those levels suggesting antiinflammatory effects of PB, which is consistent with reduction of the proinflammatory TNF-α observed in Fig. 4. PB antiinflammatory effects were also consistent with previous studies that have shown that PB exerted antiinflammatory and cellular protective effects, *ex vivo*, on pancreatic β-cells^63^. Pancreatic and liver LCA levels changed in a similar way to that in serum, while no LCA was detected in heart tissues. The reduction in LCA levels in pancreas and liver tissues is consistent with that in serum, which suggests that pancreatic and liver cellular uptake is directly proportional to LCA systemic absorption or LCA tissue metabolism is significantly influenced by the treatments. LCA levels in faeces were higher in all treated groups compared with HFD, which is consistent with its lower systemic absorption levels, except in the UDCA group due to possible feedback effects resulting in less metabolic processes and LCA production in the gut, although ileal levels were increased which suggests LCA metabolism occurring in the lower part of the gastrointestinal tract, most likely the colon. Compared with HFD, UDCA serum levels were higher, except in the M group were levels were undetected suggesting significant alteration to bile acid metabolic pathways of primary and secondary bile acids, which is consistent with M effects on CDCA and LCA serum levels. UDCA ileal levels were lowered by HFD, hence higher levels by all treatments except M group were levels were undetected, consistent with serum levels. Pancreatic levels were detected in LFD and all other groups except in HFD, M and PBUDCA groups suggesting significant disturbances of the bile acid metabolism, which is consistent with their effects on primary and secondary bile acids. The effects of M on our bile acid profile is consistent with published studies showing M reducing bile acid gut reabsorption in T2D patients^64^, with some studies suggesting M effects on bile acid profile is via ileal protein transporters^65^, reducing ileal bile salt reabsorption^66^, or influencing crosstalk processes between the nuclear bile acid receptor farnesoid X receptor and the nutrient-sensitive kinase, 5′ adenosine monophosphate-activated protein kinase^67^. UDCA heart levels were detected only in LFD and PB-L groups suggesting that HFD reduced UDCA cardiocyte cellular uptake and only PB in low dose neutralised the HFD effect. Despite the fact that there were no detectable levels for CDCA and LCA in heart tissues, and UDCA being less lipophilic than CDCA, it was present in cardiocytes after prediabetic mice were treated with PB-L possibly as a consequence of high levels of UDCA observed in serum, ileum and pancreas. Similarly, UDCA levels in the liver were reduced in the prediabetic mice with M and PBUDCA treatments lowering the levels further suggesting potential interference of M and PB treatments on bile acid regulation by hepatocytes particularly since PBUDCA microcapsules showed strong pH-targeted release (Fig. 1), PB significant uptake at the hyperglycaemic state (Fig. 2) and PB concentrations in the liver were the highest in the PBUDCA group (Fig. 3). Similar to ileal levels and compared with HFD group, UDCA faecal levels were high in LFD, PB-L, PBUDCA and UDCA groups suggesting minute metabolism within cecum and colon although PB-H treatment showed undetected UDCA levels suggesting strong local effects of high dose PB on gut-UDCA metabolism and systemic absorption, particularly when serum levels of UDCA were higher in the PB-H group compared with untreated HFD control. In general, PB treated groups showed overall decreased primary and secondary bile acids in serum, and overall increased UDCA levels in serum, suggesting reduction in cholesterol levels resulting in less bile acid synthesis compensated by higher levels of tertiary bile acids via feedback mechanisms associated with bile acid enterohepatic recirculation processes^68,69^. Hence, the highest ileal UDCA levels were observed in the UDCA group with corresponding high levels of LCA in the ileum being detected in both the M and UDCA groups. No CDCA was detected in the ileum of UDCA mice and this might be attributed to alterations in the enterohepatic cycling of endogenous bile acids caused by UDCA, increasing the formation of secondary bile acid LCA and the tertiary bile acid UDCA within the gastrointestinal tract (Table 3).

**Table 3:** Summary of the levels of bile acids among treated groups compared with control (HFD).

Treatments	Primary bile acid profile (CDCA)						Secondary bile acid profile (LCA)						Tertiary bile acid profile (UDCA)					
	serum	ileum	pancreas	heart	liver	faeces	serum	ileum	pancreas	heart	liver	faeces	serum	ileum	pancreas	heart	liver	faeces
LFD	±	±	↑	±	↓	↓	↓	↓	↓	±	↓	↑	↑	↑	↑	↑	↑	↑
M	↓	↑	±	±	↓	↓	↓	↑	↓	±	↓	↑	↓	↓	±	±	↓	↓
PB-L	↓	↓	↑	±	↓	↓	↓	↓	↓	±	↓	↑	↑	↑	↑	↑	±	↑
PB-H	↓	↑	↑	±	↓	↓	↓	↓	↓	±	↓	↑	↑	↑	↑	±	±	↓
PB-UDCA	↓	↑	±	±	↓	↓	↓	↓	↓	±	↓	↑	↑	↑	±	±	↓	↑
UDCA	↓	±	↑	±	↓	↓	↓	↑	↓	±	↓	±	↑	↑	↑	±	±	↑

±indicates no significant net effect, ↑ indicates increase and ↓ indicates decrease levels, based on Fig. 5 measurements.

Currently PB is administered orally, as tablets and is widely used for hypercholesterolemia in China and South East Asia. It comes in different strength tablets including 125 mg, with its recommended dosing of 500–1000 mg/day. Similarly, the bile acid UDCA is administered orally as tablets, and is widely used for primary liver cirrhosis, globally. It comes mainly as 250 mg tablets, with its recommended dosing of 500–1000 mg/day. PB and UDCA tablets are coated to prevent release in stomach, and the tableting formulations have been widely used for several decades.

## Conclusion

The microencapsulating method deployed was successful in producing PBUDCA targeted-delivery micro/nano capsules, which are stable, compatible and have desirable and consistent shape and delivery profile in our prediabetic mouse model. PBUDCA enhanced survival of pancreatic β-cells and muscle cells (*ex vivo*) with substrate-selectivity of PB toward the efflux protein transporters MRP1, MRP2 and MRP3, and significant PB oral absorption optimised by PBUDCA microencapsulation, in serum, liver, ileum and heart (*in vivo*); 6-months oral dosing. PBUDCA lowered blood glucose comparable to M, and exerted significant antiinflammatory effects, while anti-lipidemic effects remained insignificant. PBUDCA exerted significant bile acid modulation effects suggesting PB involvement in the enterohepatic recirculation of bile acids, although the exact molecular and cellular pathways and their influence on the bile acid synthesis and feedback mechanisms remained unclear. Future studies should aim to investigate various polymer-bile acid formulation systems to further optimise the delivery and therapeutic impact of dual antioxidant-bile acid microcapsules for the treatment of diabetes mellitus.

## Materials and Methods

### Materials and drug preparation

Probucol, metformin, low viscosity sodium alginate, ursodeoxycholic acid and sodium alginate were purchased from Sigma Chemical Co, USA, while calcium chloride from Scharlab S.L, Australia. The reagents were purchased from Merck (Australia) and were used without modifications. Stock of PB (20 mg/mL) and UDCA (4 mg/mL) were prepared by vortexing the powders with 10% gel^3,14,27–29^. Preparations were mixed for 7 hours, and used within two day of preparation.

### Microencapsulation fabrication, stability/shelf life, and *in vitro* studies

Microcapsules of PBUDCA and UDCA were prepared as established in our laboratory by Ionic Gelation Vibrational Jet Flow Technology, which utilises a Büchi encapsulator (Büchi Labortechnik, Flawil, Switzerland) under a constant liquid flow rate of 1 mL/min. The microcapsules were formed at 2% CaCl_2_ ionic gelation bath before being washed in water for a few minutes prior to collection and stability/shelf life assessed using Accelerated Stability Chambers using our well-established methods^14,27,28,30–33^. Microcapsule morphology and surface topography were examined using Micro-CT (a SkyScan 1172 A Micro-CT, Kontich, Belgium) and Zeiss-Neon 40EsB FIBSEM (USA) as per our well-established methods^29,70^. The surface characteristics were examined via FIB SEM (Zeiss Neon 40EsB, USA). Osmotic stability of the microcapsules was determined by placing 1 g of microcapsules in phosphate buffered saline for 14 days at 37 °C, and was calculated by weight gain attained compared to initial ‘dry’ weight^14,27,28^. The mechanical resistance of the microcapsules was determined by placing 200 microcapsules in a shaker and vibrating them over 14 days, and the resistance index was calculated as percentage of damaged microcapsules to intact microcapsules^30,34^. Microcapsules’ buoyancy was examined through placing 200 microcapsules in 200 mL of simulated intestinal fluids which consisted of enzyme-based phosphate buffer. The solution was stirred periodically at a set temperature 37.5 °C. The buoyancy index was calculated as the percentage of floating microcapsules^3^. The heat resistance testing was performed by incubating 200 freshly made microcapsules in a climatic chamber (Angelantoni Environmental and Climatic Test Chamber, Italy) set at 37.5 °C for 14 days. The stability index was determined mathematically by calculating the percentage of undamaged microcapsules (no change in colour, texture, appearance or structural integrity) compared to pre-incubated fresh microcapsules^3,11,14^.

### *Ex vivo* studies

NIT-1 mouse-cloned pancreatic β-cells and C2C12 mouse-cloned muscle cells were cultured separately in sterile flasks containing growth media optimised with glucose, antibiotics and amino acids using our established methods^3,14^. Viability assays were performed at two glucose concentrations (5.5 mM and 35.5 mM) over a 52 hour period^3,14^. In order to measure oxidative stress, NIT-1 and C2C12 cells were cultured using two different glucose concentrations of 5.5 and 35.5 mM for two days. Stock solutions of Dichloro-dihydro-fluorescein diacetate and azobis-2-methyl-propanimidamide Dihydrochloride were freshly prepared and stored at −20 °C and aliquots used for the antioxidant assay. After two days incubation, microplates containing treated cells were placed in an Enspire Multimode Plate Reader (PerkinElmer, USA) and the fluorescence was read after one hour. Using this method, the intensity of fluorescence directly corresponds to the formation of fluorescent oxidised radical species dichlorofluorescein. The lower the fluorescence reading, the greater the antioxidant activity conferred^71^. The cellular antioxidant assay was done in triplicates and data was normalised for viable cell count. In order to examine UDCA and PB cellular uptake from the microcapsules, at normoglycaemic glucose levels (5.5 mM) and hyperglycaemic glucose levels (35.5 mM), the cells were treated with UDCA or PBUDCA microcapsules for 48 hours, then microcapsules removed and cells washed with PBS, sonicated to rupture, and washed with ice-cold acetonitrile. Similar conditions were used to examine effects of M, G or BA inhibitory effects of PB cellular uptake. The supernatants were removed and analysed using a liquid-chromatography/mass spectroscopy (LC-MS) instrumentation that involved a flow rate of 0.25 mL/min using methanol-water as 65:35 mixture with assay run times of 15 minutes per run based on our established methods^42,49,72,73^. Since UDCA was endogenously produced, PB cellular permeation rate was measured using our well-established methods^74^ and selected ABC-transporters substrates were incorporated with PB and UDCA to examine transporters’ selectivity to PB and UDCA.

### *In vivo* studies

Six-week old, wild type (C57BL/6J) male mice were attained from the Animal Resources Centre (Australia). Mice were randomly allocated into seven groups, 10 each (n = 70). Group-1 was given low fat diet (LFD; healthy) and empty microcapsules, group-2 was given high fat diet (HFD; insulin-resistance) and empty microcapsules, group-3 was given HFD and metformin (200 mg/kg/day), group-4 was given HFD and low dose PB (80 mg/kg/day), group-5 was given HFD and high dose of PB (800 mg/kg/day), group-6 was given HFD and PBUDCA microcapsules (PB: 80 mg/kg/day and UDCA 70 mg/kg/day) and group-7 was given HFD and UDCA microcapsules (70 mg/kg/day). HFD consisted of AIN93M rodent chow enriched in 30% (w/w) lard, 0.5% (w/w) cholesterol and 15% (w/w) fructose (Specialty Feeds, Perth, Australia).

All mice were maintained on half-day dark cycle (22 °C) and with water and food *ad libitum*. At the end of 6-months experiment, mice were anaesthetized with isoflurane and euthanised by cardiac puncture followed by cervical dislocation. Blood was collected into EDTA tubes and stored on ice. Plasma was separated by short-speed centrifugation at 4 °C and stored at −80 °C. Tissues of different organs were removed at stored in 4% paraformaldehyde (PFA) at −80 °C. The animal experiments were approved by Curtin University Animal Ethics Committee and all experiments were performed according to the Australian Code of Practice for the care and use of animals for scientific purposes.

## Probucol and Bile Acids Analysis

### PB HPLC analysis

Standard concentrations and quality control samples of PB in mobile phase acetonitrile: water were prepared for the range of 0.04 to 0.8 mg/ml. volume of injection was 10 µL per injection. Shimadzu HPLC Prominence was used, and consisted of Shimadzu LC-20AT liquid chromatographer, SIL-20A autosampler and SPD-20A-UV/Vis detector (Japan). 160 μL of mobile phase (acetonitrile: water in a 96:4% v/v ratio) was added to 40 µL of purified plasma and vortex-mixed for 5 seconds and centrifuged at 15000 RPM for 15 minutes. Twenty µL of the supernatant was removed and transferred to autosampler vials ready for analysis^20^.

### Bile acids’ LC-MS analysis

Blood, tissue and fecal bile acids analyses were carried out via liquid chromatography mass spectrometry (LCMS). In brief, LCMS (Shimadzu LCMS 2020 system, Shimadzu Corporation, Kyoto, Japan) included a Phenomenex C18 column (Phenomenex Corporation, Torrance, California, USA) 10 cm in length and 2 mm in diameter and with 5 µm particle size. The flow rate set at 0.25 mL/min and the mobile phase was methanol (65%) and water (35%) at pH 2.9, with the standards and quality control samples being within the range of 1–1000 ng/ml. The analysed bile acids CDCA, LCA and UDCA had retention times of 2.6, 5.1 and 1.5 minutes respectively, with a flow rate of 1.5 L/min using our well-established methods^18,42,72,73^.

### Biological analysis

Blood glucose levels were measured via tail vein venepuncture using Accucheck (Roche Laboratories, Switzerland) and HbA1c measurements were via Siemens DCA Vantage Analyser (Siemens Healthcare Diagnostics, New York, USA). Plasma cholesterol and triglycerides were assessed via enzymatic assays (Randox Laboratories, Crumlin, UK)^75^, while NEFAs were assessed with NEFA-C (ASC-ACOD method, Osaka, Japan)^76^. Visceral fat depositions were examined visually at the end of experiment, while plasma cytokines were assessed using cytokine bead array kit (BD Biosciences, California, USA) via Attune Acoustic Focusing Flow Cytometer (Life Technologies, Carlsbad, California, USA) as per our established methods^18,20,77^.

### Statistical analysis

Values are expressed as means ± standard error of the mean. Statistical measurements were carried out using parametric/non-parametric analysis or using a one way ANOVA and a Tuckey post-hoc, as appropriate. GraphPad Prism Version X8.2 (GraphPad, USA) was utilised for p value analyses.
